# SIRT1 activation mediates heat-induced survival of UVB damaged Keratinocytes

**DOI:** 10.1186/s12895-017-0060-y

**Published:** 2017-06-10

**Authors:** Leslie Calapre, Elin S. Gray, Sandrine Kurdykowski, Anthony David, Pascal Descargues, Mel Ziman

**Affiliations:** 1 School of Medical Science, Edith Cowan University, 270 Joondalup Drive, Joondalup, Perth, WA 6027 Australia; 2 GENOSKIN Centre Pierre Potier, Oncopole, Toulouse, France; 3 School of Pathology and Laboratory Medicine, University of Western Australia, Crawley, WA Australia

**Keywords:** Heat stress, UVB, Keratinocytes, Apoptosis, p53, SIRT1, p53 deacetylation

## Abstract

**Background:**

Exposure to heat stress after UVB irradiation induces a reduction of apoptosis, resulting in survival of DNA damaged human keratinocytes. This heat-mediated evasion of apoptosis appears to be mediated by activation of SIRT1 and inactivation of p53 signalling. In this study, we assessed the role of SIRT1 in the inactivation of p53 signalling and impairment of DNA damage response in UVB *plus* heat exposed keratinocytes.

**Results:**

Activation of SIRT1 after multiple UVB *plus* heat exposures resulted in increased p53 deacetylation at K382, which is known to affect its binding to specific target genes. Accordingly, we noted decreased apoptosis and down regulation of the p53 targeted pro-apoptotic gene *BAX* and the DNA repair genes *ERCC1* and *XPC* after UVB *plus* heat treatments. In addition, UVB *plus* heat induced increased expression of the cell survival gene *Survivin* and the proliferation marker Ki67. Notably, keratinocytes exposed to UVB *plus* heat in the presence of the SIRT1 inhibitor, Ex-527, showed a similar phenotype to those exposed to UV alone; i.e. an increase in p53 acetylation, increased apoptosis and low levels of *Survivin*.

**Conclusion:**

This study demonstrate that heat-induced SIRT1 activation mediates survival of DNA damaged keratinocytes through deacetylation of p53 after exposure to UVB *plus* heat

**Electronic supplementary material:**

The online version of this article (doi:10.1186/s12895-017-0060-y) contains supplementary material, which is available to authorized users.

## Background

The incidence of skin cancers, particularly of keratinocyte-derived cancers, basal cell carcinoma (BCC) and squamous cell carcinoma (SCC), has increased in the last few decades [1, 2]. Chronic exposure to UV radiation, predominantly UVB, is the most common cause of these cutaneous malignancies [3, 4]. Studies have shown moreover, that high temperature can increase the rate of tumour formation in mice and potentiate the carcinogenic effects of UV [5, 6] and thus, heat stress may also be a risk factor in skin carcinogenesis. Given this evidence, the paucity of studies on the consequences of repeated exposure to both high temperatures and UVB on epidermal cell biology requires addressing [7].

A few studies have shown that exposure to heat stress prior to UVB irradiation protected human and murine keratinocytes against DNA damage [8–10]. The heat-mediated reduction of DNA damage in the form of photoproduct formation, particularly cyclobutane pyrimidine dimers (CPDs), was suggested to be a consequence of increased expression and pre-activation of heat shock proteins (HSPs), thought to diminish the lethality of UV radiation on keratinocytes [9, 11–13]. However, previous studies in our laboratory showed UVB and UVB *plus* heat exposures induce the same level of CPD formation. Conversely, UVB *plus* heat treated samples had a significantly reduced number of apoptotic keratinocytes when compared to cells treated with UVB alone [14].

The reduction in apoptosis and thus the survival of DNA damaged keratinocytes in UVB *plus* heat treated samples was thought to be associated with the presence of phosphorylated Sirtuin 1 (SIRT1) and inactivation of p53 signalling [14]. Since SIRT1 induces deacetylation of the p53 protein [15–17], we hypothesised that SIRT1 activation by heat stress affects the ability of p53 to regulate downstream gene targets. Thus, UVB *plus* heat treated keratinocytes are unable to mediate an adequate cellular stress response to UVB mediated DNA damage.

SIRT1 is an NAD-dependent histone deacetylase that is known to be increased by heat stress [18, 19], but inhibited by UVB [20]. Its activity is vital for the maintenance of chromosomal integrity and control of various cellular processes including cell metabolism and cellular stress response [21, 22]. The deacetylase activity of SIRT1 regulates several stress-induced transcription factors including p53 [15, 23] and HSF1 [18, 19, 24].

Of particular interest to our study is the ability of SIRT1 to deacetylate the lysine 382 residue of the p53 protein [24–26]. SIRT1-mediated deacetylation of p53 reduces its DNA binding capability, leading to deregulation of expression of p53 dependent genes and impairment of the tumour suppressor functions of the protein [15, 27]. Such functions include the regulation of cell proliferation, DNA damage repair, cell cycle arrest, and apoptosis [28–30]. It appears then that heat-mediated activation of SIRT1 may affect the efficiency of repair of UVB–induced DNA damage and inhibit apoptosis. Survival of DNA damaged cells, that otherwise would have undergone apoptosis, results in the accumulation of UVB-induced mutation in dividing cells [31].

In this study, we aimed to determine whether activation of SIRT1 is the main biomolecular mechanism driving the heat-mediated survival of DNA damaged cells in UVB *plus* heat treated keratinocytes. In particular, we investigated the effects of SIRT1 activation on the on-set of the p53-mediated cellular stress response, approximately 4 h after UV irradiation in normal skin [32, 33], and the efficiency of this system to mediate repair and/or apoptosis in UVB *plus* heat treated keratinocytes harbouring DNA damage. We used ex vivo skins and in vitro primary keratinocytes to assess this, as well as whether UVB *plus* heat leads to the increased presence of unrepaired CPDs, and survival and proliferation of keratinocytes despite DNA damage. Finally, we assessed whether blocking SIRT1 activity abrogates the evasion of apoptosis and cell survival observed after UVB *plus* heat exposures.

## Methods

### Ex vivo skin model

Thirty-two NativeSkin® (Genoskin, France) ex vivo skin models, taken from non-sun exposed skin of healthy donors, are punch biopsies of normal human skin embedded in a matrix and fixed in a cell culture insert. Informed consent was obtained from donors, and commercialisation and experimental use of the skin biopsies were approved by the Comité de Protection des Personnes (CPP, France) and the ECU Human Research Ethics Committee.

### NHEK

Primary adult human epidermal keratinocytes (NHEK-c, Promocell) were cultured in vitro using Keratinocyte Growth Medium 2 (Promocell, Germany) supplemented with bovine pituitary extract (0.004 ml/ml), recombinant human epidermal growth factor (0.125 ng/ml), recombinant human insulin (5 μg/ml), hydrocortisone (0.33 μg/ml), epinephrine (0.39 μg/ml), human transferrin holo (10 μg/ml), CaCl_2_ (0.06 mM) and penicillin/streptomycin (Sigma-Aldrich, Australia).

### UVB radiation and heat exposure

Skins and primary keratinocytes were exposed to heat stress and UVB *plus* heat according to previous protocols [14]. Samples were then analysed for DNA damage, apoptosis and gene expression at 4 h after the last exposure. UVB and/or heat treated samples were also evaluated for apoptosis and expression of Ki67 2 days post-exposures. For SIRT1 inhibition experiments, 1 μM of Ex-527 was added to the cell culture medium of NHEKs prior to exposures. Each experiment was performed in triplicate and each set of experiments included untreated cells which underwent similar handling.

### Immunocytochemistry and Immunohistochemistry

Primary keratinocytes were seeded in a 12-well plate at 100,000 cells/well in LabTek Chambered -Microscopic slides (Thermofisher, Australia) and exposed to UV and/or heat stress. For immunocytochemistry, cells were fixed with 4% paraformaldehyde, washed twice with TBS. Skin tissues were processed and stained according to previous protocols [14]. Primary NHEK and skin sections were incubated with antibodies to either thymine dimer (CPD) (mouse monoclonal, 1:500 dilution; Kamiya Biomedical, USA), p53 antibody (rabbit monoclonal 1:50 dilution; Abcam, USA), p53 acetyl K382 (rabbit monoclonal 1:200, Abcam USA), SIRT1, SIRT1-p, Casp-3 (cleaved), Survivin or anti-pan Cytokeratin and ki67 (rabbit polyclonal, 1:50 dilution; Abcam, USA). The mouse monoclonal antibody to pan-cytokeratin (1:50; Abcam, USA) was used to label keratinocytes. The secondary antibodies anti-mouse Alexa Fluor 488 (for keratin and CPD) and anti-rabbit Alexa Fluor 550 (for caspase 3, p53 and ki67) were used for detection. Three sections were analysed per exposure replicate and five images of randomly selected fields-of-view were captured from each section. To determine percentages of keratinocytes positive for each individual antibody, positive cells were quantified within 5 images randomly chosen per section.

### Western blot

Cytosolic and nuclear proteins were isolated from untreated and treated primary keratinocytes using AllPrep RNA/Protein Kit (Qiagen, Australia) according to manufacturer’s instructions. A total of 20 μg protein was loaded onto Mini-PROTEAN TGX Stain-Free Precast Gels (Bio-Rad, USA) and then transferred onto Midi Trans-Blot 0.2 μm nitrocellulose membrane (Bio-Rad). The membrane was probed with rabbit monoclonal p53 antibody or p53 acetyl K382 and detected using Clarity Western ECL Blotting Substrate detection kit (Bio-Rad) as per the manufacturer’s instructions. The blot was visualised using the ChemiDoc Touch system (Bio-Rad) and protein levels were assessed and quantified relative to standards using ImageLab software (Bio-Rad).

### Apoptosis

In the ex vivo skin models, apoptosis was quantified via the percentage of CPD keratinocytes with active caspase 3 (casp-3), a marker of apoptosis [34]. The level of apoptosis for exposed NHEKs was determined using Annexin V-FITC Apoptosis Detection Kit I (BD Pharmingen, USA) and cells were stained as per the manufacturer’s instructions. Samples were analysed using the Gallios™ flow cytometer (Beckman-Coulter). For each sample, 10,000 events were acquired. Annexin V^+^PI^−^cells represented early apoptotic populations and Annexin V^+^PI^+^ cells represented either late apoptotic or secondary necrotic populations.

### Ex vivo gene expression analysis

RNA was isolated from skin tissues and cells using an AllPrep RNA/DNA Mini Kit (Qiagen). The quality of the RNA extracted from samples was analysed using an Agilent RNA 6000 Bioanalyser Kit. Differential expression of *BAX*, *Survivin* (*BIRC5*), *ERCC1* and *XPC* genes in the UV and/or heat exposed samples were verified by quantitative real-time PCR (qRT-PCR) using the ViiA 7 Real-Time PCR System (Applied Biosystems, USA).

Quantitative RT-PCR was performed based on the manufacturer’s instructions using TaqMan probes (Life Technologies, Australia) for *BAX*, Survivin (*BIRC5*), *ERCC1* and *XPC* genes. Human 18S (Taqman, Life Technologies) was used as the endogenous reference gene. Relative quantification of the expression levels of each transcript in each sample were calculated using the Delta-Delta CT method relative to untreated controls.

### Statistical analyses

Two-way ANOVA was used to analyse differences across treatment groups, while parametric unpaired t-tests were used to detect differences between specific treatment groups in all experimental categories, with *p*-values <0.05 considered significant.

## Results

### UVB *plus* heat induced SIRT1 phosphorylation and decreased apoptosis of DNA damaged keratinocytes

**Fig. 1:**
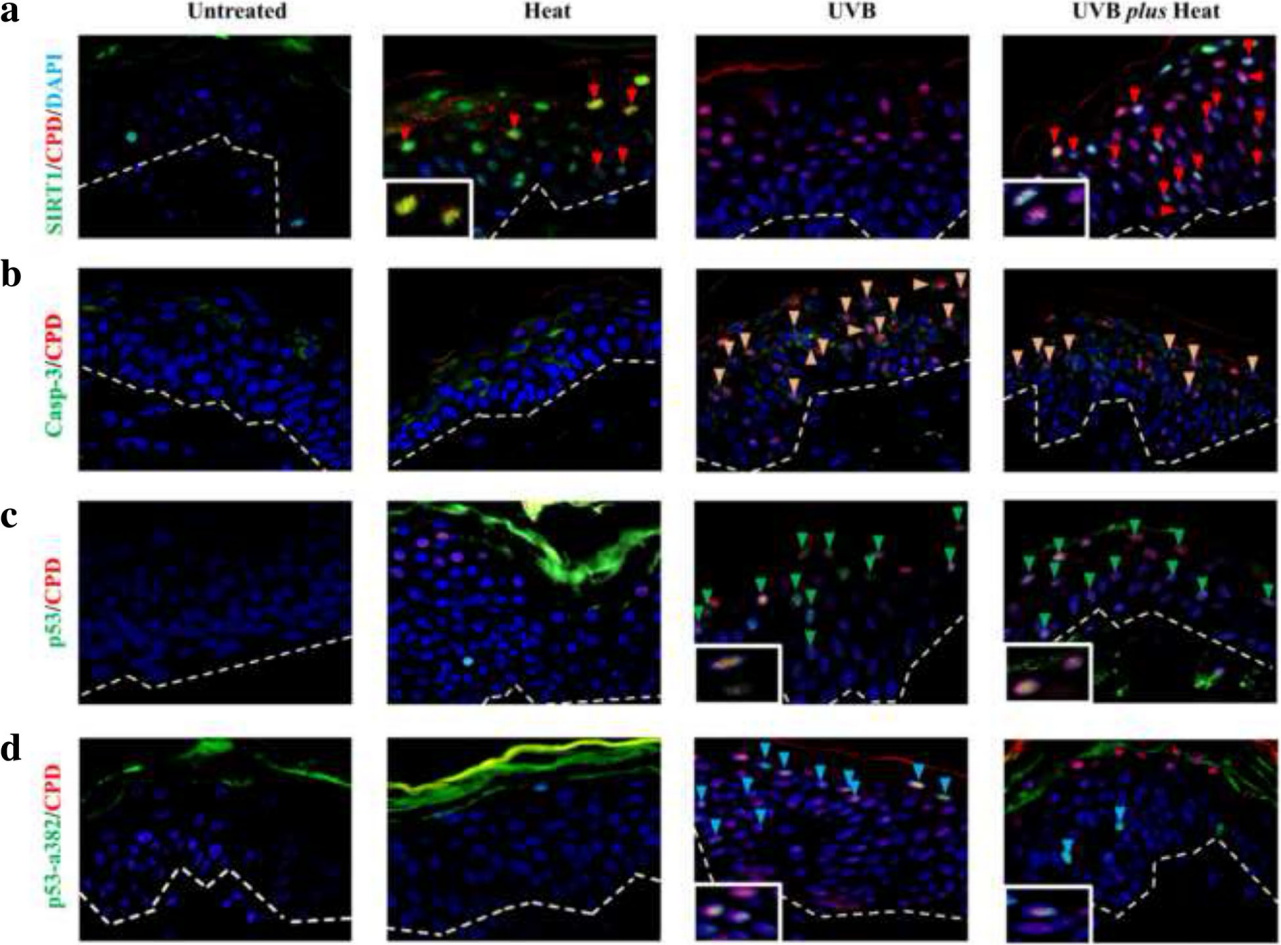
Exposure to repeated UVB plus heat significantly decreased acetylated p53 levels in keratinocytes. Representative immunohistochemical staining of ex vivo skin either untreated, or exposed to heat, UVB or UVB plus heat, for nuclear DNA (DAPI, *blue*), CPD (*red*) and (**a**) phosphorylated SIRT1 (SIRT1-p), (**b**) caspase (casp-3), (**c**) total p53 or (**d**) acetylated p53 (p53-a382). Inset images are an enlarged view of cells positive for CPD and SIRT1 (*red arrows*), CPD and Casp-3 (*orange arrows*), CPD and p53 (*green arrows*) or CPD and p53-a382 (*blue arrows*). Broken lines denote the epidermal/dermal border. All images are at 400X magnification

We first determined whether activation of SIRT1 is apparent after UVB *plus* heat exposure. Ex vivo skin models and normal primary human epidermal keratinocytes (NHEK) in vitro were exposed to UVB and/or heat once a day for 4 consecutive days. Cells that harboured DNA damage signatures (CPD^+^) and activated SIRT1 (SIRT1-p^+^) were quantified after the last exposure in samples treated with UVB and/or heat relative to untreated controls (Fig. 1a and Additional file 1: Figure S1a).

The percentage of keratinocytes containing CPDs was high in both UVB irradiated (84 ± 7%) and UVB *plus* heat (79 ± 3%) treated skin models (Table 1), and in NHEKs exposed to UVB (91 ± 8%) or UVB *plus* heat (87 ± 3%). UVB *plus* heat treated keratinocytes were observed to contain both SIRT1-p and CPD positive staining in skin models (50 ± 3%) and in vitro NHEKs (55 ± 3%). Notably, neither of the models had SIRT1-p staining in cells irradiated with UVB alone (Fig. 1a, Additional files 1 and 2: Figure S1a and Table S1). SIRT1 activation, however, was also apparent in samples treated with heat alone, clearly linking SIRT1 activation to heat exposure.

**Table 1:** Effect of UVB and/or heat exposure in keratinocytes of the ex vivo skin models or NHEK in vitro

*Percentage Mean ± S.D.*				
	Untreated	Heat	UVB	UVB *plus* Heat
*Skin*				
DNA Damaged Cells (%)	0 ± 0	5 ± 3	84 ± 7	79 ± 3
Apoptotic Cells (%)	0 ± 0	14 ± 1	37 ± 3	15 ± 4***
SIRT1-p^+^/CPD^+^ (%)	0 ± 0	32 ± 2	0 ± 0	50 ± 3
Apoptotic Cells (%)	0 ± 0	14 ± 1	37 ± 3	15 ± 4***
p53^+^/CPD^+^ (%)	0 ± 0	0 ± 0	76 ± 3	69 ± 3
p53-ace382^+^/CPD^+^ (%)	0 ± 0	0 ± 0	79 ± 6	40 ± 2**
*NHEK*				
DNA Damaged Cells (%)	0 ± 0	2 ± 1	91 ± 8	87 ± 3
Apoptotic Cells (%)	2 ± 1	3 ± 1	25 ± 3	7 ± 4
SIRT1-p^+^/CPD^+^ (%)	1 ± 1	34 ± 5	0 ± 0	55 ± 3**
p53^+^/CPD^+^ (%)	0 ± 0	0 ± 0	84 ± 3	79 ± 2
p53-ace382^+^/CPD^+^ (%)	0 ± 0	0 ± 0	75 ± 4	28 ± 7**

Statistically significant differences relative to UVB: ** = *p*-value ≤0.001 or *** = *p*-value ≤0.0001

*SIRT1-p* ^*+*^ */CPD* ^+^ cells positively stained for SIRT1-p and CPD

*p53* ^+^ */CPD* ^+^ cells positively stained for p53 and CPD

*P53-ace382* ^+^ */CPD* ^+^ cells positively stained for p53-acetylated (lys382) and CPD

UVB *plus* heat treated samples also exhibited a significantly decreased percentage of DNA damaged keratinocytes that were apoptotic (Casp-3/CPD positive cells) compared to those irradiated with UVB alone in ex vivo skin (15 ± 4% vs 37 ± 3%, *p* = 0.004) and in NHEKs (7 ± 4% vs 25 ± 3%, *p* = 0.0003) (Fig. 1b and Table 1). Altogether, these results suggest that SIRT1 phosphorylation is activated in response to repeated UVB *plus* heat exposures and this is associated with evasion of apoptosis within CPD damaged-keratinocytes.

### UVB *plus* heat induced deacetylation of p53

The p53 protein is a key regulator of cellular response to stress. The p53 regulation of DNA damage response and cell cycle arrest is coordinated via acetylation and phosphorylation [23, 35]. It has previously been shown that SIRT1 induces deacetylation of p53 [36]. Thus, we determined the percentage of cells expressing total p53 and acetylated p53 (p53-ace382) after repeated UVB and UVB *plus* heat exposure.

There were no significant differences in the percentage of CPD positive cells expressing p53 between UVB and UVB *plus* heat treated keratinocytes ex vivo or in vitro (Fig. 1c, Additional file 1: Figure S1b and Table 1). However, a significantly lower percentage of CPD positive keratinocytes had acetylated p53 in UVB *plus* heat treated samples compared to those irradiated with UVB alone in ex vivo models (40 ± 2% vs 79 ± 6%, *p* = 0.002) and in NHEKs (28 ± 7% vs 75 ± 4%, *p* = 0.001) (Fig. 1d and Additional file 1: Figure S1c). Notably, there was an inverse relationship between the percentage of CPD positive cells expressing SIRT1-p and those with acetylated p53 in the UVB and UVB *plus* heat treated samples. More importantly, our results demonstrated that repeated exposure of keratinocytes to UVB *plus* heat did not prevent the expression of p53 in these cells, but appeared to affect the acetylation status of the p53 protein.

### UVB *plus* heat induces deregulation of p53 downstream target genes

**Fig. 2:**
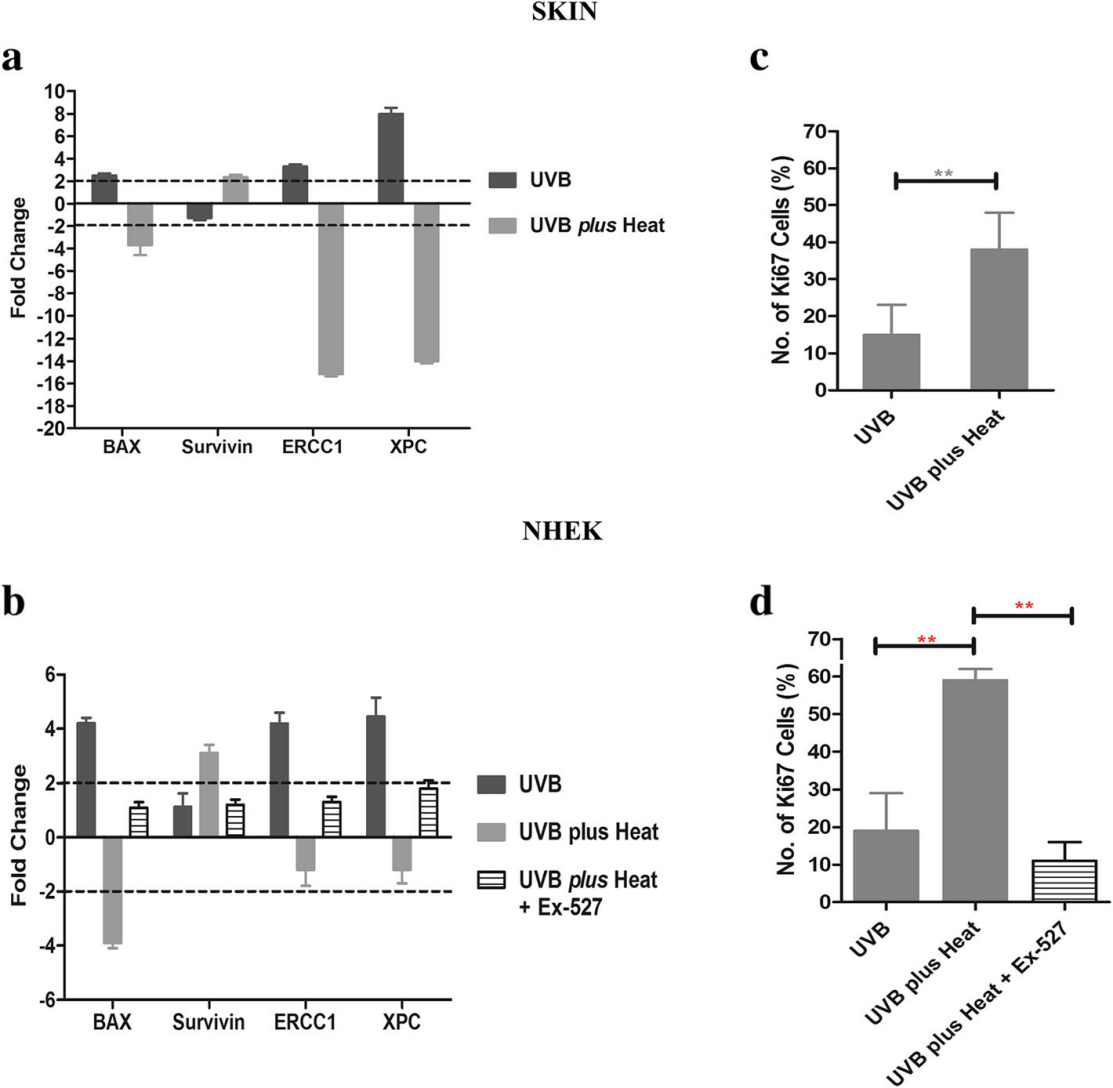
Effect of UV and/or heat exposure on p53 downstream gene targets and cell proliferation. **a-b** Fold change on mRNA expression of BAX, Survivin, ERCC1 or XPC in keratinocytes of the (**a**) skin and (**b**) in vitro, relative to untreated controls. **c-d** Bar graphs of the percentage mean (+/− SD) of keratinocytes positive for ki67 per field of view either (**c**) in ex vivo skin or (**d**) in vitro. Statistically significant differences are indicated with ** *p* < 0.001 and/or *** for *p*-values *p* < 0.0001

SIRT1-mediated deacetylation of the p53 protein is known to reduce its DNA binding capability, leading to deregulation of expression of p53 dependent genes [15, 37]. We therefore, measured the expression levels of downstream target genes of p53 that may be affected by deacetylation. We first looked at gene expression levels of *BAX*, a regulator of cell apoptosis and *Survivin* (*BIRC5*), a regulator of cell survival, as we previously observed these genes to be affected by UVB *plus* heat treatment of keratinocytes at 48 h after exposure [14]. We found that UVB irradiation caused significant upregulation of *BAX*, but not of *Survivin*, in the ex vivo skins models (Fig. 2a) and in NHEKs (Fig. 2b). By contrast, UVB *plus* heat exposure induced downregulation of *BAX* and significant upregulation of *Survivin* in both the skin and NHEKs.

We then examined the gene expression of *XPC* and *ERCC1*, which are p53-regulated nucleotide excision repair (NER) genes. *XPC* and *ERCC1* are commonly upregulated in UVB irradiated cells and are necessary for the repair of UVB-induced CPD damage [38]. UVB irradiated keratinocytes in ex vivo skin models and in vitro showed significant upregulation of *ERCC1* (3- and 4-fold) and *XPC* (9- and 4-fold) in skin and NHEKs respectively. Notably, this upregulation was not apparent in UVB *plus* heat exposed NHEKs (Fig. 2b), and in fact, *ERCC1* and *XPC* were significantly downregulated in UVB *plus* heat treated skin models (Fig. 2a). These results confirm that exposure to UVB *plus* heat stress appears to compromise the p53-mediated DNA repair mechanisms within keratinocytes.

### UVB *plus* heat increased ki67 expression in DNA damaged keratinocytes

Next we quantified the percentage of cells that were positive for ki67, a protein commonly used as a marker of proliferation [39–42]. To ensure that cells were provided sufficient time for repair and mitosis and that the effects on repair and proliferation were persistent, quantification of proliferative (ki67-positive) keratinocytes was conducted 2 days after the last exposure. UVB *plus* heat treatment caused a significant increase in the percentage of keratinocytes that were positive for ki67 relative to UVB treated samples in ex vivo skins (36 ± 8% vs 16 ± 10%, *p* = 0.0427) and in vitro (59 ± 3% vs 19 ± 2%, *p* = 0.003) (Fig. 2c-d, Additional file 3: Figure S2)*.* These results show that heat, presumably as a result of the impaired p53-mediated cellular stress response, leads to increased survival and proliferation of keratinocytes.

### SIRT1 inhibition reduces UVB *plus* heat-induced inactivation of p53 signalling

To determine whether inhibition of SIRT1 will reverse the pro-survival effects of heat exposure on UVB damaged keratinocytes, we treated NHEKs with Ex-527, a known inhibitor of SIRT1. Previous studies have shown that exposure to Ex-527 effectively reduces SIRT1 activity [43–45], and produces maximal p53 acetylation, but does not induce significant toxicity in untreated keratinocytes at a dose of 1 μM [4, 46].

**Fig. 3:**
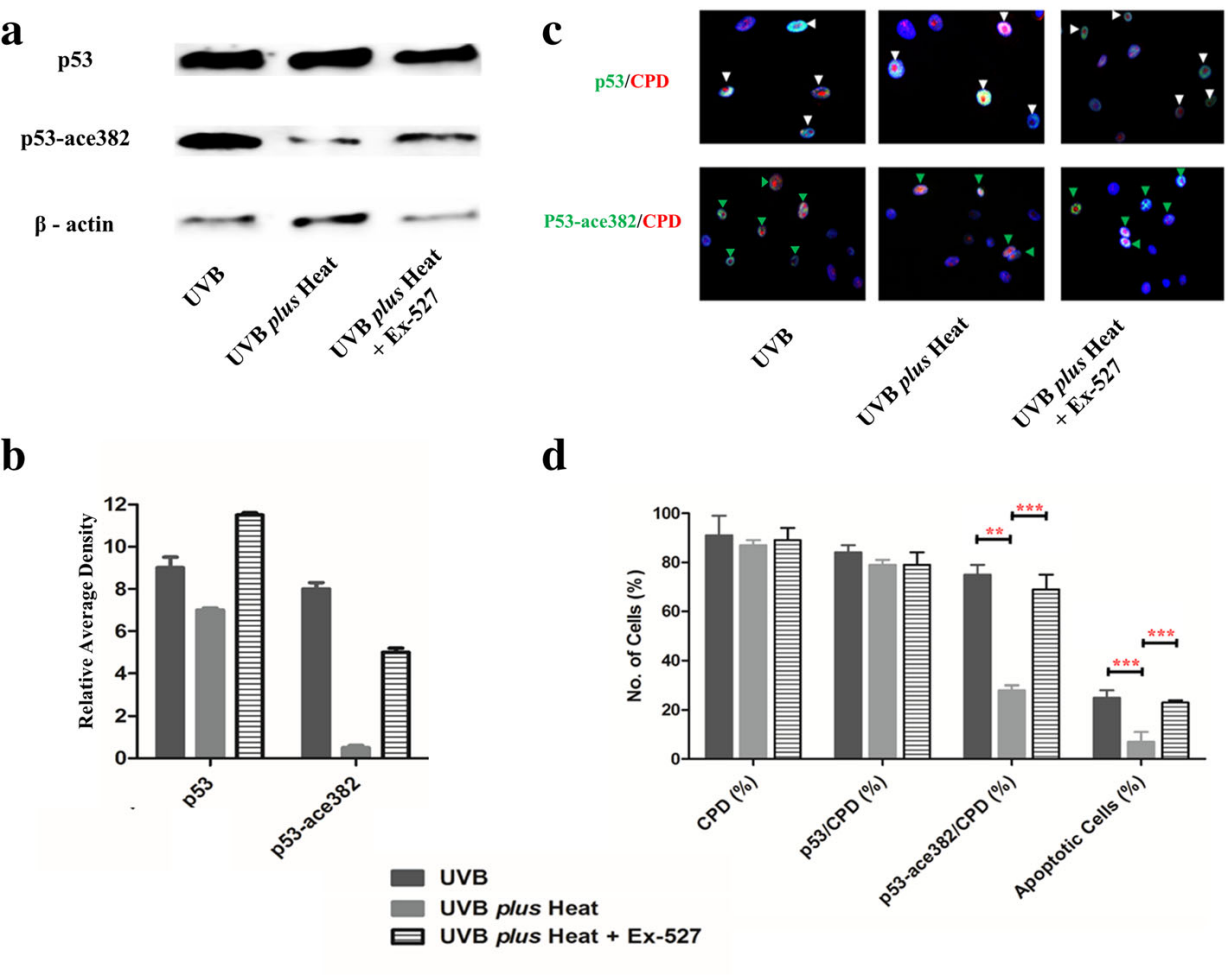
Inhibition of SIRT1 in UVB plus heat significantly increased acetylated p53 protein expression and apoptosis of keratinocytes in vitro. **a** Immunoblot showing levels of total p53, and acetylated p53 protein in UVB, UVB plus heat and UVB plus heat with the SIRT1 inhibitor (Ex-527). **b** Quantification of protein levels by relative average density standardised by β-actin. **c** Representative immunohistochemical staining of cells positive for CPD and p53 (*white arrows*), CPD and p53-a382 (*green arrows*) in primary keratinocytes exposed to UVB or UVB plus heat with or without SIRT1 inhibitor Ex-527. All images are at 400X magnification. **d** Bar graphs of percentage mean (+/− SD) of DNA damaged (CPD), apoptotic, p53 or p53-a382 positive primary keratinocytes exposed to UVB or UVB plus heat with or without Ex-527. Statistically significant differences are indicated with ** for *p* < 0.001 and/or *** for *p*-values *p* < 0.0001 respectively

Keratinocytes that were treated with UVB *plus* heat, in the presence of 1 μM of Ex-257, had significantly higher levels of acetylated p53 protein in comparison with UVB *plus* heat treated samples, resembling the levels of acetylated p53 found in cells treated with UVB alone (Fig. 3a-b). Accordingly, protein expression analysis by immunohistochemistry showed that the percentage of cells that were positive for both CPD and acetylated p53 was significantly increased in UVB *plus* heat treated keratinocytes in the presence of Ex-257 (69 ± 4% vs 28 ± 7%, *p* = 0.0001), compared to exposed cells not treated with the SIRT1 inhibitor (Fig. 3c-d). It is important to note, that the percentage of p53-a382/CPD keratinocytes after UVB *plus* heat together with Ex-527 was comparable to those in samples irradiated with UVB alone (69 ± 4% and 75 ± 4% respectively). Moreover, the presence of SIRT1 appears to correspond to downregulation of *BAX* and the upregulation of *Survivin* observed in UVB *plus* heat treated keratinocytes (Fig. 2b). These results clearly indicate that SIRT1 is important in driving heat-mediated survival of UVB-damaged keratinocytes. However, SIRT1 inhibition did not restore the upregulation of *BAX*, *XPC* and *ERCC1* to levels observed in samples treated with UVB alone. This result suggests that these genes may have returned to baseline levels or might be influenced by factors additional to SIRT1 and/or p53.

### SIRT1 inhibition diminished UVB *plus* heat effects on apoptosis and proliferation of keratinocytes

Since UVB *plus* heat causes a significant decrease in apoptosis in keratinocytes, we then determined if SIRT1 inhibition reverses this effect. Less than 5% of untreated NHEKs with or without Ex-527 were apoptotic (Additional file 4: Figure S3). However, the presence of Ex-527 significantly increased cell apoptosis from 7 ± 4% to 23 ± 1% (*p* = 0.004) in UVB *plus* heat, which is similar to levels found in UVB irradiated samples (25 ± 3%) (Fig. 3b). In addition, at 2 days post exposure, in the presence of SIRT1-inhibitor there was a significant decrease in the proportion of keratinocytes that were ki67 positive in UVB *plus* heat treated samples (Fig. 2d and Additional file 3: Figure S2b). Thus, survival and proliferation of DNA damaged cells appears to be ameliorated by the presence of the SIRT1 inhibitor.

## Discussion

In this study, we clearly confirm that the exposure of keratinocytes to UVB *plus* heat impairs the p53-mediated cellular stress response. We also show that this phenomenon is a consequence of heat-induced SIRT1 activation in these cells. SIRT1 activation appears to induce post-translational modifications of the p53 protein, affecting the ability of this protein to regulate the expression of downstream gene targets important for regulating DNA damage repair and apoptosis. As a result, keratinocytes containing UVB-induced DNA damage are able to survive and proliferate.

SIRT1 is essential for maintaining chromatin stability but can affect the function of other transcription factors, including HSF-1 and p53, by deacetylation of these protein [26, 47, 48]. In the case of HSF-1, SIRT1-mediated deacetylation of this protein increases its DNA-binding ability, leading to increased expression of heat shock proteins [18, 19, 49]. Conversely, SIRT1-mediated deacetylation of p53 diminishes the ability of this protein to bind to the promoters of its target genes, particularly those required for apoptosis and cell cycle arrest [25, 36, 50].

This study confirmed that SIRT1 is indeed a key molecular event in the UVB *plus* heat-mediated effects on keratinocyte biology. In line with previous observations that SIRT1 activation is inhibited by UV radiation but increased in response to heat stress [19, 24, 26], we observed increased SIRT1 phosphorylation in heat treated but not in UV irradiated samples. The combination of UV *plus* heat also resulted in SIRT1 activation. However, the exact mechanism behind heat-induced activation of SIRT1 is not yet known, and this needs to be assessed in order to fully understand the role of SIRT1 in UVB *plus* heat-mediated survival of DNA damaged keratinocytes.

Interestingly, the presence of the SIRT1 inhibitor (Ex-527) prevented the UVB *plus* heat-mediated survival of DNA damaged keratinocytes. Furthermore, inhibition of SIRT1 in UVB *plus* heat treated keratinocytes induced a significant increase in p53 acetylation and activation of p53 signalling, leading to increased cell apoptosis and a decrease in proliferative keratinocytes. Thus, SIRT1 appears to be indispensable for evasion of apoptosis and survival of UVB *plus* heat treated keratinocytes harbouring DNA damage. By means of post-translational modifications to the p53 protein and consequently, inactivation of p53 signalling, heat-mediated SIRT1 activation appears to negate the ability of UVB *plus* heat keratinocytes to mount an appropriate response to UVB-induced DNA damage.

Despite the significant impact of SIRT1 activation on p53 signalling, increased levels of phosphorylated SIRT1 were found to have no effect on the levels of the p53 protein, i.e. there were no significant differences in the level of p53 protein in UVB or UVB *plus* heat treated samples. Thus, the diminished efficiency of p53-mediated cellular stress response in UVB *plus* heat treated samples does not appear to arise from changes in p53 protein transcription nor its translocation to the nucleus. Therefore, lack of an appropriate p53-mediated DNA damage response in keratinocytes after UVB *plus* heat exposure appears to be a consequence of SIRT1-imposed post-translational modifications to the p53 protein.

The interference of the p53-mediated surveillance of DNA damage via deacetylation appears to be one of the primary effects of UVB plus heat exposure in keratinocytes. One of the most notable consequences of SIRT1-induced deacetylation and therefore, inactivation of p53 in UVB plus heat treated keratinocytes, was the significant downregulation of ERCC1 and XPC, which are responsible for the recognition and repair of CPDs [51–53], as the products of these genes are responsible for the recognition of distortions in the DNA helix and activation of global genome nucleotide excision repair pathways (GG-NER) [54, 55]. In particular, the *XPC* promoter contains a putative p53 response element and elevated XPC expression after UV irradiation occurs in a p53-dependent manner [56–58]. Thus, the persistence of CPD could be a result of the lack of effective recognition of the existing damage, preventing the removal of CPDs and resulting in impaired clearance of DNA lesions. This finding is of particular importance considering a recent study showed that a significant percentage of ‘normal’ epidermal keratinocytes harbour UV-signature mutations in genes that are key drivers of squamous cell carcinoma [59]. By affecting the ability of keratinocytes to recognise and repair DNA damage, consecutive exposure to UVB plus heat stress may induce further accumulation of mutations in these ‘normal’ cells, fuelling their transformation into a malignant phenotype. However, a more comprehensive study on the effects of UVB plus heat-mediated SIRT1 activation on the mediators of the nucleotide excision repair system, including XPA, is necessary to provide an improved understanding of the effects of SIRT1 deacetylation on the nucleotide excision repair mechanisms overall. More importantly, further studies are required to determine whether heat stress can indeed exacerbate UVB-induced skin carcinogenesis.

It is important to note that the survival mechanism we observed in our UVB *plus* heat treated keratinocytes are in direct contradiction to previous reports. A few studies have shown that prior heat treatment protects human and murine keratinocytes against UVB-induced DNA damage, as a consequence of pre-activated heat shock proteins [9, 11–13]. However, the mechanisms involved in our experiments, where heat is added after UVB, appear to be primarily driven by heat-mediated post-translational modifications to the p53 protein. Nevertheless, heat shock response mediators, particularly HSP90, were found upregulated in UVB *plus* heat treated keratinocytes at 4 h post exposure (data not shown), suggesting that the heat shock response is functional despite repeated exposure to multiple stressors. Given the importance of HSPs in resisting UVB-induced apoptosis, UVB *plus* heat treatment therefore, provides keratinocytes with additional capacity to survive and proliferate.

Interestingly, in the presence of SIRT1 inhibitor (Ex-527), UVB *plus* heat treated cells did not show similar upregulated levels of HSP90 expression in vitro and in ex vivo skin (data not shown). Thus, SIRT1 activation appears to be required for the full induction of the heat shock response. A similar observation indicating that SIRT1 can affect the regulation of the heat shock response has also been reported previously [19]. In their study, Westerheide and colleagues observed diminished HSP90 expression when HeLa cells were exposed to nicotinamide, another Sirt1-inhibitor drug. In addition, they found that SIRT1 deacetylates HSF-1, increasing its DNA binding affinity to the promoters of HSPs. This result indicates that SIRT1 may act as an upstream regulator of the HSF-1-mediated heat shock response, and thus elucidates the importance of this particular Sirtuin protein in thermal stress response. However, the exact mechanism as to how heat induces SIRT1 activation needs to be fully determined.

It is also important to note that while we found evidence to show that UVB *plus* heat induces survival of DNA damaged keratinocytes, these observations are that of a UVB followed by heat exposure model. Heat stress and UVB radiation occur simultaneously in the environment. Thus, activation of SIRT1, and its subsequent effects on the p53-mediated cellular stress response and the heat shock response, must be confirmed in a UV and heat concurrent exposure model. Nonetheless, we propose that heat in the environment would similarly affect UVB irradiated cells, i.e. it will aid in the survival of DNA damaged keratinocytes.

## Conclusions

In conclusion, our study uncovered a survival pathway intrinsically induced by UVB *plus* heat exposure and mediated by SIRT1 activation. In addition, we provide additional evidence that exposure to high temperatures, subsequent to UV irradiation, impairs effective cell arrest, DNA repair and/or apoptosis of DNA damaged keratinocytes, indicating that subsequent UV and heat may potentially act synergistically to create pre-cancerous lesions. However, translational studies using mouse models may be required to determine whether heat treatment will enhance UVB-induced tumourigenesis.

## Additional files

Additional file 1: Figure S1. Exposure to UVB plus heat significantly decreased acetylated p53 levels in keratinocytes. Immunohistochemical staining of nuclear DNA (DAPI, blue), CPD (red) and active caspase (casp-3), phosphorylated SIRT1 (SIRT1-p), total p53 or acetylated p53 (p53-a382) (green) in primary keratinocytes (NHEK) in vitro either untreated, or exposed to heat, UV or UVB plus heat. (TIFF 1482 kb)

Additional file 2: Table S1. Keratinocytes expressing SIRT1 only after UVB and/or heat exposure. (DOCX 12 kb)

Additional file 3: Figure S2. Exposure to UVB plus heat induced an increase in the number of keratinocytes expressing Ki67. (a) Immunohistochemical staining of nuclear DNA (DAPI, blue), Ki67 (red) and cytokeratin (CytoK) (green) in UV or UVB plus heat treated skin. (b) Fluorescent immunocytochemistry staining of nuclear DNA (DAPI, blue) and Ki67 (green) in primary keratinocytes (NHEK) in vitro. (TIFF 1702 kb)

Additional file 4: Figure S3. SIRT1 inhibitor (Ex-527) does not induce toxicity to NHEK. Levels of cell apoptosis in untreated keratinocytes with or without Ex-527. (TIFF 243 kb)

## Abbreviations

CPD: Cyclobutane pyrimidine dimers
NHEK: Primary normal human epidermal keratinocytes
UVB: Ultraviolet radiation B
