# Supplementary figures and images for: SIRT1 activation mediates heat-induced survival of UVB damaged Keratinocytes
Source: BMC Dermatol. 2017 Jun 10;17:8. doi: 10.1186/s12895-017-0060-y (PMC5466784; doi:10.1186/s12895-017-0060-y)

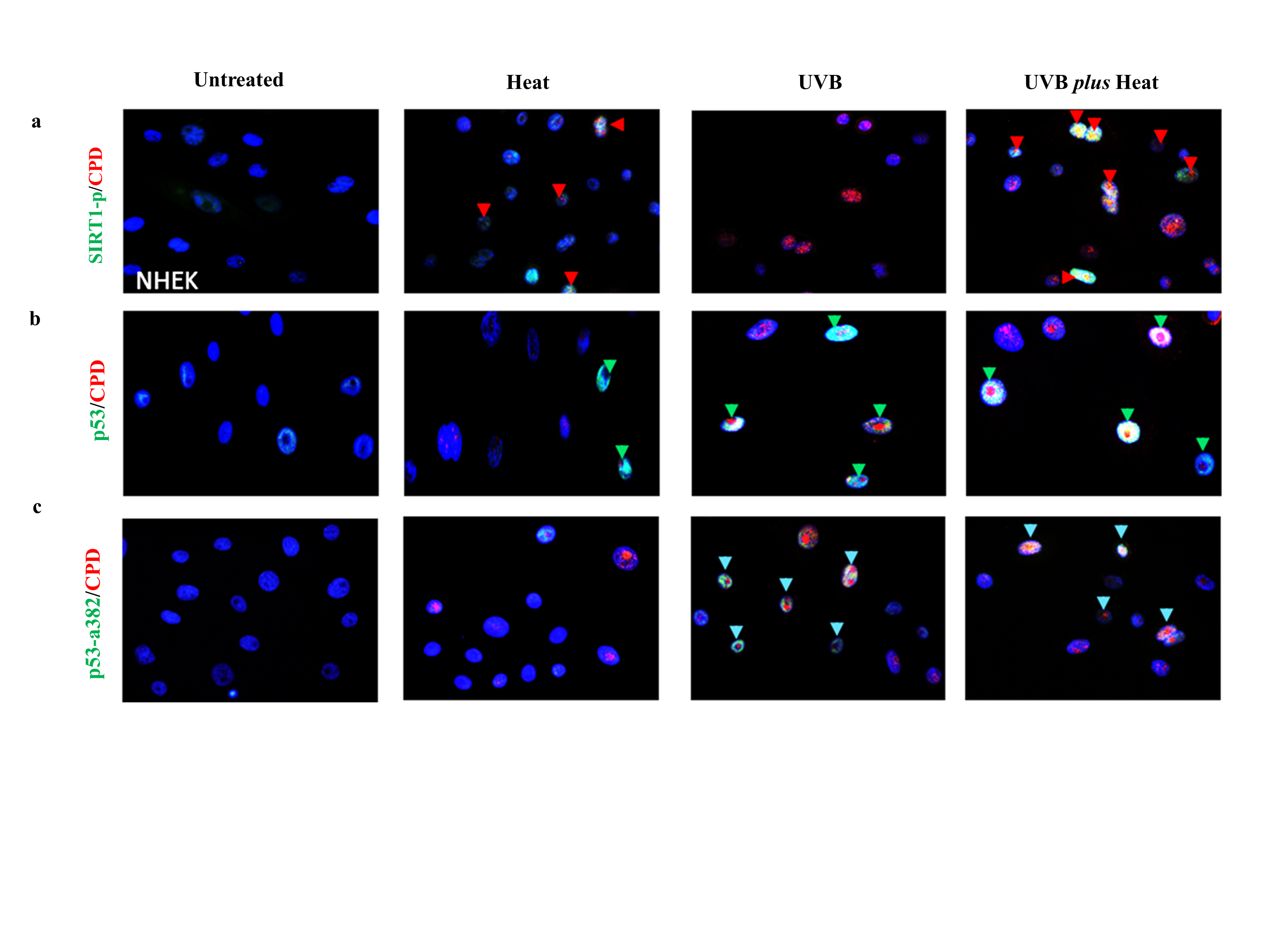

Supplement: Supplementary file 1 — Exposure to UVB plus heat significantly decreased acetylated p53 levels in keratinocytes. Immunohistochemical staining of nuclear DNA (DAPI, blue), CPD (red) and active caspase (casp-3), phosphorylated SIRT1 (SIRT1-p), total p53 or acetylated p53 (p53-a382) (green) in primary keratinocytes (NHEK) in vitro either untreated, or exposed to heat, UV or UVB plus heat. (TIFF 1482 kb) [file 12895_2017_60_MOESM1_ESM.tif]

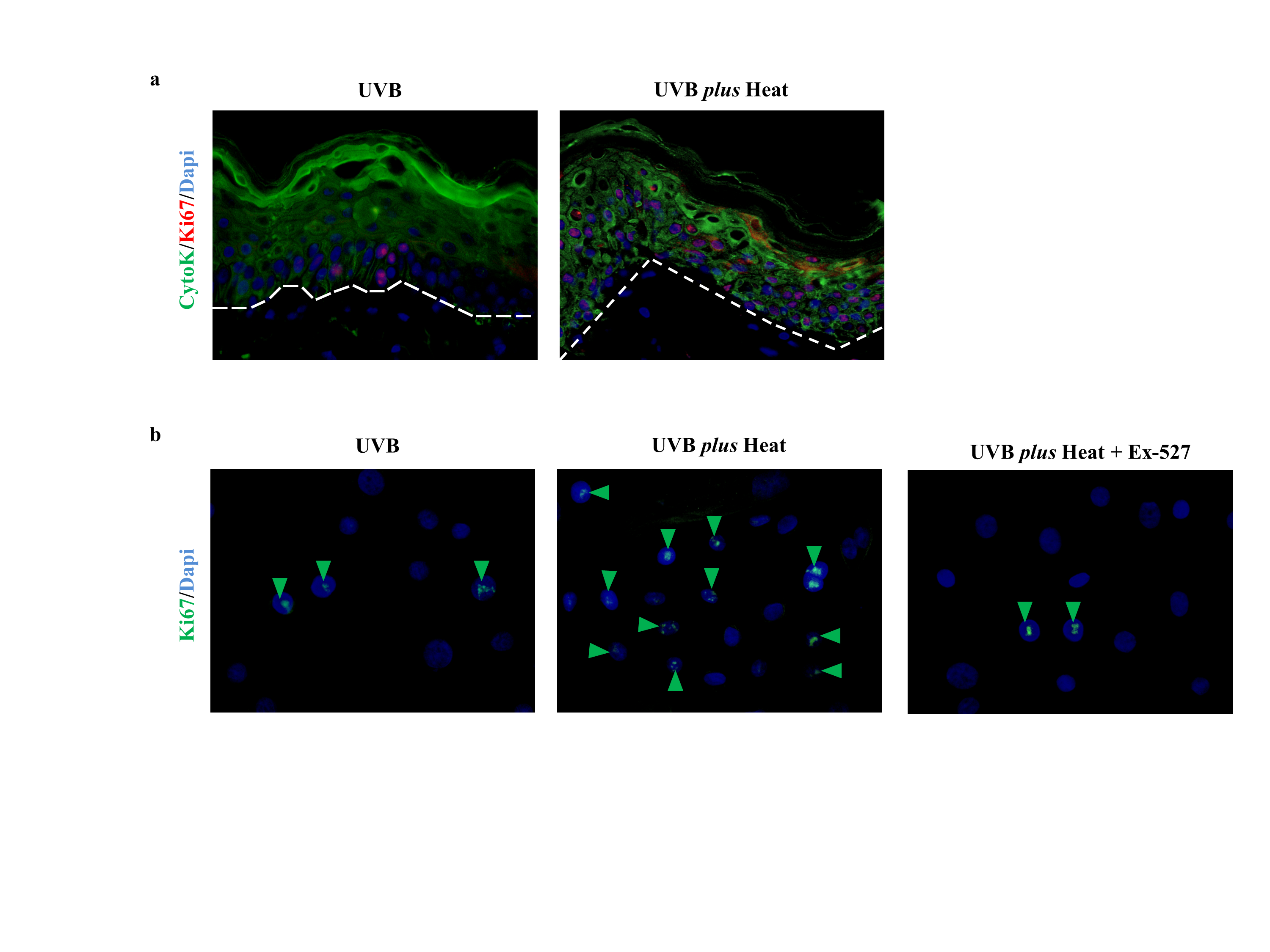

Supplement: Supplementary file 3 — Exposure to UVB plus heat induced an increase in the number of keratinocytes expressing Ki67. (a) Immunohistochemical staining of nuclear DNA (DAPI, blue), Ki67 (red) and cytokeratin (CytoK) (green) in UV or UVB plus heat treated skin. (b) Fluorescent immunocytochemistry staining of nuclear DNA (DAPI, blue) and Ki67 (green) in primary keratinocytes (NHEK) in vitro. (TIFF 1702 kb) [file 12895_2017_60_MOESM3_ESM.tif]

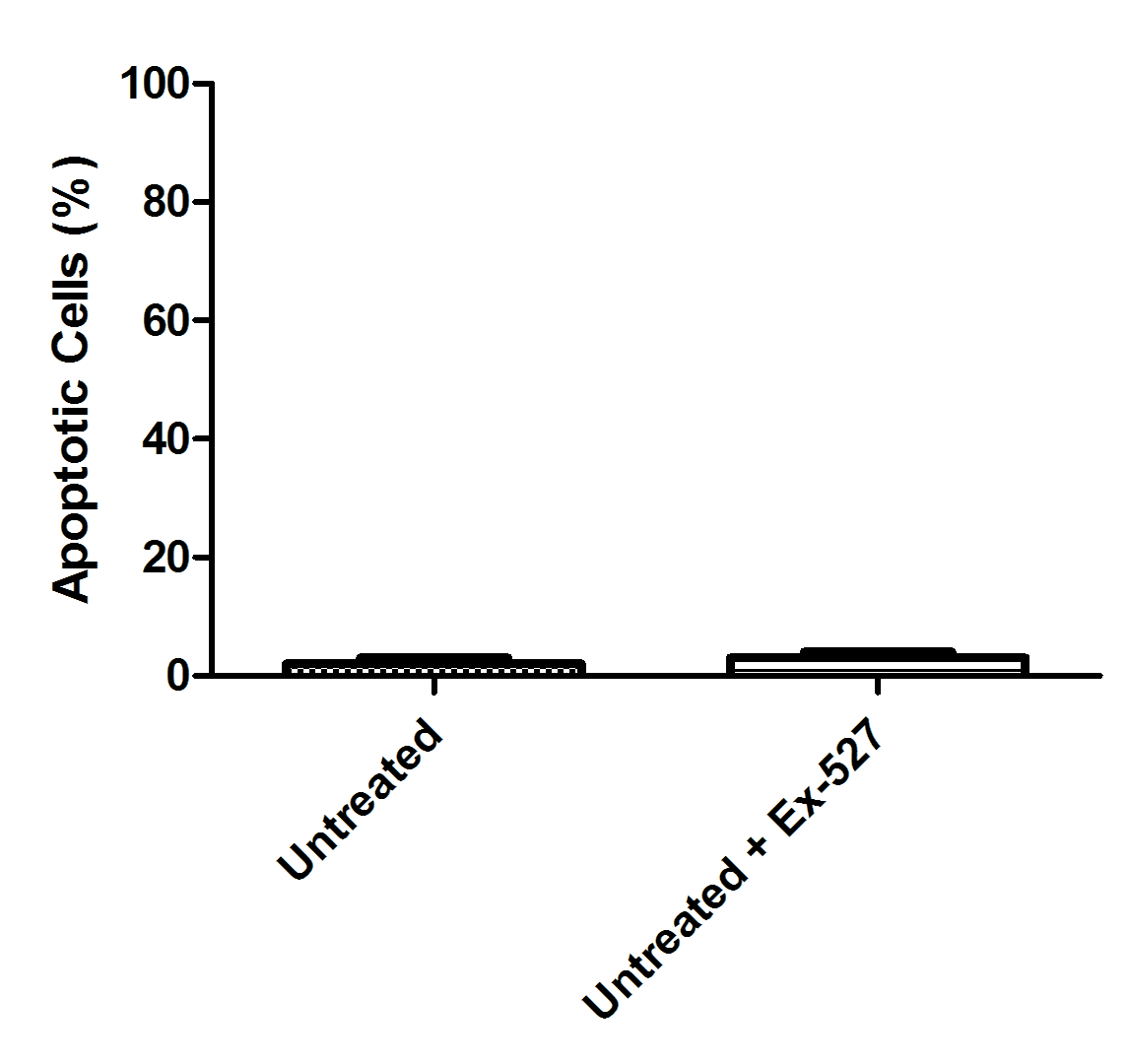

Supplement: Supplementary file 4 — SIRT1 inhibitor (Ex-527) does not induce toxicity to NHEK. Levels of cell apoptosis in untreated keratinocytes with or without Ex-527. (TIFF 243 kb) [file 12895_2017_60_MOESM4_ESM.tif]
