# Supplementary material for: SIRT1 activation mediates heat-induced survival of UVB damaged Keratinocytes
Source: BMC Dermatol. 2017 Jun 10;17:8. doi: 10.1186/s12895-017-0060-y (PMC5466784; doi:10.1186/s12895-017-0060-y)
Supplement: Supplementary file 2 — Keratinocytes expressing SIRT1 only after UVB and/or heat exposure. (DOCX 12 kb) [file 12895_2017_60_MOESM2_ESM.docx]

**Table S1: Keratinocytes expressing SIRT1 only after** **UVB and/or heat exposure.**

| *Percentage Mean ± S.D.* | | | | | |
| --- | --- | --- | --- | --- | --- |
|  | **Untreated** | | **Heat** | **UVB** | **UVB *plus* Heat** |
| *Skin* | | | | | |
| SIRT1-p^+^ | | 0±0 | 19±2 | 0±0 | 56±6 |
| *NHEK* | | | | | |
| SIRT1-p^+^ | | 1±1 | 30±5 | 0±0 | 49±3 |
| *Number of SIRT1*^+^ *keratinocytes relative to total number of cells.* | | | | | |
